# *Epilepsia Open*—October 2026 Announcements

**DOI:** 10.1002/epi4.70360

**Published:** 2026-10-19

## ILAE CONGRESSES

Curso virtual: Correlación anátomo‐electro‐clínica

12 October–20 November 2026

Cápsula virtual sobre terapia cetógena en epilepsia farmacoresistente 2026

19–25 October 2026

8th East Mediterranean Epilepsy Congress & 14th Saudi Epilepsy Society Congress

29–31 October 2026

Al Khobar, KSA

16th Asian & Oceanian Epilepsy Congress

19–22 November 2026

Kuala Lumpur, Malaysia

## 2027

16th ILAE School on Pre‐Surgical Evaluation for Epilepsy and Epilepsy Surgery

11–15 January 2027

Brno, Czech Republic

20th Latin American Summer School on Epilepsy

22–27 February 2027

Medellín, Colombia

37th International Epilepsy Congress

28 August to 1 September 2027

Amsterdam, Netherlands

## 2028

17th European Epilepsy Congress

23–27 September 2028

London, United Kingdom

## WEBINARS

Project ECHO ‐ University of Nebraska Medical Center

10 October 2026

What every SEEG brain mappers need to know beyond the spikes

15 October 2026

When Behaviour Becomes the Main Problem: A Multidisciplinary Approach to the Management of Psychiatric Comorbidities in DEEs

19 October 2026

ILAE‐Africa webinaire en français

20 October 2026

Genomics – when, why and how?

ILAE e‐Forum Series 2026

21 October 2026

From Ideas to Impact: Nursing Innovation in Action

28 October 2026

Patient education and counselling: A look beyond seizure control

15 November 2026

Nursing Care for Children with Epilepsy in Slovakia and the Czech Republic

23 November 2026

Neuroimaging – what, why and when

ILAE e‐Forum Series 2026

25 November 2026

## OTHER CONGRESSES

2026 CLAE Annual Scientific Meeting

2–4 October 2026

Niagara‐on‐the‐Lake, Ontario, Canada

Behind the Curtain: A Multidisciplinary Approach to Epilepsy Care

7 October 2026

Harrogate, UK

ILAE British Branch 2026 Annual Scientific Conference

8–10 October 2026

Harrogate, UK

Cleveland Clinic International Epilepsy Summit 2026

8–11 October 2026

Independence, Ohio, USA

XII Congreso de la Sociedad Española de Epilepsia

15–17 October 2026

Córdoba, Spain

43ra Conferencia Epilepsia del Caribe

17 October 2026

Carolina, Puerto Rico

Annual Meeting of the German‐Austrian‐Swiss Epilepsy Working Groups 2026

27–30 October 2026

Vienna, Austria

5th Video‐EEG in Paediatric Epilepsies

29–31 October 2026

Madrid, Spain

Epilepsy Society of Australia 40th Annual Scientific Meeting

4–6 November 2026

Melbourne, Victoria, Australia

4th Advanced Course on the Pharmacological Treatment of Drug‐Resistant Epilepsies

6–8 November 2026

Palma de Mallorca, Spain

EPIBAQ 2026

12–14 November 2026

Barranquilla, Colombia

1st Swiss Neuro Week

18–20 November 2026

Bern, Switzerland

ILAE British Branch Clinical Epilepsy Course for Doctors in Training & Allied Healthcare Professionals

21 November 2026

Birmingham, UK

20th Asian Epilepsy Surgery Congress

22–23 November 2026

Kuala Lumpur, Malaysia

43rd International Conference on Advances in Psychiatry and Mental Health

7–8 December 2026

Dubai, United Arab Emirates

Encephalitis 2026

7–8 December 2026

London, UK & Online

## 2027

5th European Congress of Neurology and Neuropsychiatry (NEURO 2027)

22–23 February 2027

Rome, Italy

European Workshop on Developmental and Behavioural Outcome Measures in Developmental and Epileptic Encephalopathies (DEE)

25–27 February 2027

Leuven, Belgium

14th Tri‐National Meeting of the German and Austrian Societies for Epileptology and the Swiss Epilepsy League

11–13 March 2027

Basel, Switzerland

Fourth International congress on Structural Epilepsy & Symptomatic Seizures

7–9 April 2027

Gothenburg, Sweden

International Conference on Technology and Analysis of Seizures 2027

23–27 August 2027

Newcastle upon Tyne, United Kingdom

